# Medical Items

**Published:** 1893-07

**Authors:** 


### *MEDICAL ITEMS.*

---

Dr. George M. Sternberg has been appointed surgeon-general of the army to succeed Dr. Sutherland, retired.

---

Medical Inspector J. Rufus Tryon has been appointed surgeon-general of the navy, to succeed Dr. J. Mills Browne, who retired in May.

---

Dr. C. B. Penrose has been elected professor of gynecology in the University of Pennsylvania, in the place of Dr. William Goodell, resigned.

---

Dr. Bernard Wolff, formerly of Virginia, has located in Atlanta and will confine his practice to diseases of the skin. He will lecture on Dermatology in the Southern Medical College.

---

Before the North Carolina examining board in May thirty-two out of seventy-nine applicants for license failed to pass the examinations. We may yet hear from some of these in Georgia.

---

We clip the following "quack ad." from a Georgia daily paper: "Dr. J. J. S.—Permanently located. In the specialties venereal. Lost energy restored. Female irregularities and poison oak. Cure guaranteed. Address," etc.

---

A Georgia man recently died from reading patent medicine advertisements. He found on reading them that he had the symptoms of every ill that flesh is heir to, and becoming convinced of his inability to survive such a weight of affliction as was impending, he died from sheer hypochrondria.—*Daily paper.*

THE JOURNAL is much indebted to Dr. and Mrs. J. B. S. Holmes, of Rome, for their kind invitation to be present at the "twentieth anniversary of their wedding, Monday evening, June 26th." It would have given us much pleasure to be present. We beg Dr. and Mrs. Holmes to accept our hearty congratulations, with the wish that they will enjoy many more happy anniversaries.

---

THE third annual meeting of the American Electro-Therapeutic Association will be held in Chicago September 12, 13 and 14, at Apollo Hall, Central Music Hall Block. Members of the medical profession interested in electro-therapeutics are cordially invited to attend. AUGUSTIN H. GOELET, M. D., President.

MARGARET A. CLEAVES, M. D., Secretary.

---

FIVE thousand little graves are dug each year in Philadelphia for little babies, and 5,000 little headstones are yearly set up over their graves, all due to deaths traceable to the diseases which spring from wrong feeding. In the overwhelming majority of instances the poor food of which the babies die is bad milk, diseased milk, or skimmed milk.—*Annals of Hygiene.*

---

THE new medical department of Johns Hopkins University will be opened October 28, 1893. A four-year course will be required. Applicants for matriculation must have had preliminary training in physics, chemistry and biology, and, besides, be able to read French and German and elementary Latin. In the first year anatomy, including normal histology, physiology and physiological chemistry, will be taught. Anatomy will be continued in the second year, with pharmacology, general pathology, bacteriology and the general principles of medicine and surgery. In the last two years clinical instruction will begin in medicine, surgery, obstetrics and gynecology and the special branches. Practical work in the laboratories will also be required. The fees will be two hundred dollars per year. Both men and women will be admitted.

---

### RECEIVED, READ AND "REFUSED."

To steal is dishonest, to cheat your fellowman is dishonest, to lie for gain is dishonest; yet, there is another road to dishonesty equally as reprehensible as either of the above, viz.: To receive and read a journal, for years, and then when called upon to pay for same, after having promised to do so, or even having acted in acquiescence with published requests to notify the journal if same was not wanted, to then go to the postmaster (without having nerve enough to write to the journal) and have him forward to the office a "refused" card, is as dishonest as stealing that much direct from the pocket of the proprietors of such journal. In fact, it is worse. It is a betrayal of confidence, is a sneaking and villainous trick of which no gentleman will be found guilty; and any man who will do such a thing would steal from his grandmother's ghost. If a subscriber is not able to pay, then it is quite another thing, and in all such instances he should so inform the proprietors of the publication, instead of having the P. M. mark on the cover of the journal "refused," and also send the proprietor a card marked "refused." All men who thus attempt to beat publishers should be held up to the public to be seen in the true light. This would be an unpleasant task, but we feel equal to the emergency.—*Texas Health Journal.*

